# Geometric morphometric investigation of craniofacial morphological change in domesticated silver foxes

**DOI:** 10.1038/s41598-021-82111-9

**Published:** 2021-01-28

**Authors:** Timothy M. Kistner, Katherine D. Zink, Steven Worthington, Daniel E. Lieberman

**Affiliations:** 1 Department of Human Evolutionary Biology, Harvard University, Cambridge, MA 02138 USA; 2 Institute for Quantitative Social Science, Harvard University, Cambridge, MA 02138 USA

**Keywords:** Ecology, Evolution

## Abstract

To test the effects of domestication on craniofacial skeletal morphology, we used three-dimensional geometric morphometrics (GM) along with linear and endocranial measurements to compare selected (domesticated) and unselected foxes from the Russian Farm-Fox Experiment to wild foxes from the progenitor population from which the farmed foxes are derived. Contrary to previous findings, we find that domesticated and unselected foxes show minimal differences in craniofacial shape and size compared to the more substantial differences between the wild foxes and both populations of farmed foxes. GM analyses and linear measurements demonstrate that wild foxes differ from farmed foxes largely in terms of less cranial base flexion, relatively expanded cranial vaults, and increased endocranial volumes. These results challenge the assumption that the unselected population of foxes kept as part of the Russian Farm-Fox experiment are an appropriate proxy for ‘wild’ foxes in terms of craniofacial morphology and highlight the need to include wild populations in further studies of domestication syndrome to disentangle the phenotypic effects of multiple selection pressures. These findings also suggest that marked increases in docility cannot be reliably diagnosed from shape differences in craniofacial skeletal morphology.

## Introduction

Artificial selection has long been used to model and study evolutionary change under controlled conditions. One of the most insightful artificial selection studies is the ongoing Russian Farm-Fox (RFF) Experiment, which involves directional selection for docility in silver foxes (*Vulpes vulpes*)^1^. Here, we focus on the RFF experiment’s relevance to craniofacial features associated with domestication syndrome, in which selection for docility in domesticated taxa is accompanied by a characteristic suite of behavioral, physiological and morphological traits that are absent in their wild counterparts^2^. These features typically include curved tails, floppy ears, increased frequency of non-seasonal estrus cycles, altered levels of neurotransmitters, and retention of juvenile behaviors into adulthood^3–6^. Also included are several craniofacial features notably, shorter snouts, wider faces, smaller brains, and reductions in tooth size^2,3,7^.

The RFF experiment started in 1959 by subjecting a population of foxes (30 M, 100 F) from neighboring fur farms to intense selection for docility. Only foxes that manifested affiliative behavior (e.g., whining and tail-wagging) towards a human experimenter were allowed to breed. Approximately 5% of males and 20% of females per generation were bred in the “domesticated” group, which was periodically outbred with foxes from other Siberian fur farms to prevent drift and founder effects and ensure low inbreeding coefficients^1^. Substantial behavioral changes, including whining and tail-wagging during human contact, were observed in the “domesticated” group by 8–10 generations, and morphological changes such as floppy ears and curled tails soon followed^8^.

Despite extensive research into the genetic, physiological and soft tissue alterations in the RFF experiment^4^, only one study has investigated cranial morphological changes between unselected and domesticated foxes. According to this study, the width and height of the cranial vault were marginally smaller (~ 1% or 0.5 mm decrease) in the domesticated foxes of both sexes while bi-zygomatic width was marginally larger (~ 2% or 1.3 mm increase) in the domesticated males^9^. These findings have been used to suggest that craniofacial differences between wild and domestic counterparts, such as shorter, wider faces, smaller cranial vaults, reduced facial sexual dimorphism and overall paedomorphic crania, accompany selection for docility^2,8,10^. Further support for this hypothesis comes from evidence of craniofacial paedomorphosis in many dog breeds even though paedomorphosis by itself fails to account for all the craniofacial changes in dog domestication^11^. These features in bonobos and humans have also been invoked as evidence for self-domestication^12–15^.

There are two potential problems with the hypothesis that domestication syndrome causes craniofacial morphological change. First, inferences that observed craniofacial similarities among domesticated mammals result from selection for docility are largely based on one analysis of the RFFs that used a small number of linear measurements without correcting for the potential confounding effects of size on shape^9^. Second, as Lord and colleagues have pointed out, the unselected RFFs that were used as the control group were assumed to have wild-type craniofacial morphologies^7^. However, these foxes had already been farmed for over 60 years, first in Canada and then Russia^4^, and may have previously experienced selection pressures that altered their cranial morphology relative to their wild progenitors. Because of these issues, we test the hypothesis that craniofacial shape differs less between the unselected and domesticated populations than between either of these farmed populations and the wild fox population from which they originated.

To test this hypothesis, we took 3D landmark coordinates along with linear and endocranial volume measurements on the skulls of three groups of foxes: domesticated and unselected foxes from the RFF experiment, and wild foxes from eastern Canada and Maine, the wild population from which the progenitors of the RFFs originated. After performing geometric morphometric analyses on the 3D landmark coordinate data, we used tangent space coordinates to estimate average differences among the three populations using a Procrustes ANOVA. In addition, we analyzed linear and endocranial volume data using generalized least squares models.

## Materials and methods

### Samples

We sampled 73 adult fox skulls (*Vulpes vulpes*) from three separate sample groups: wild (8 F, 12 M), unselected (15 F, 8 M), and domesticated (15 F, 15 M). Domesticated and unselected skulls from the RFF experiment were generously provided by Dr. Trut and transported to Harvard in 2004. Unfortunately, we do not know how these foxes were chosen, but have no reason not to assume that they were selected randomly from both populations. Wild fox skulls in the study were sampled from the collections of the Museum of Comparative Zoology, Harvard University. All but two wild foxes were trapped in Canada east of Quebec between 1894 and 1952, with the majority (70%) between 1894 and 1900 (Table S1). We excluded from the study sample all juvenile skulls, as determined through lower third molar eruption and fusion of the cranial suture between the basioccipital and basisphenoid^16^, and those skulls that had evidence of damage or disease. After applying these exclusion criteria, we arrived at our final sample of 73 skulls.

### 3D landmarks

To prevent movement during measurement, each skull was embedded in styrofoam and secured to the workspace desk before 3D coordinates of 29 landmarks, listed, defined and displayed in Table S2 and Fig S1, were collected on the left half of each skull by a single analyst (TMK). Of these 29 landmarks, 17% are on the cranial base, 27% are on the neurocranium, and 55% are on the face. 3D landmark coordinates were measured with a Microscribe G2 (Positional Accuracy ± 0.38 mm, Revware, Inc.). This machine consists of a mobile robotic arm tipped with a probe. After calibration, the probe tip is placed on each landmark to record its XYZ coordinates. To avoid having to move the skulls during measuring and to limit the number of variables in our final GM analysis (too high a number may be a problem given our small sample size), we restricted landmark measurements to one half of each skull. We assume that the fluctuating asymmetry between each fox population is negligible and stable as has previously been shown in comparisons across a domestic-wild hybridization zone in mice^17^. In most cases, landmark positions were lightly marked with pencil to ensure proper probe placement.

### Linear and endocranial volume measurements

**Figure 1:**
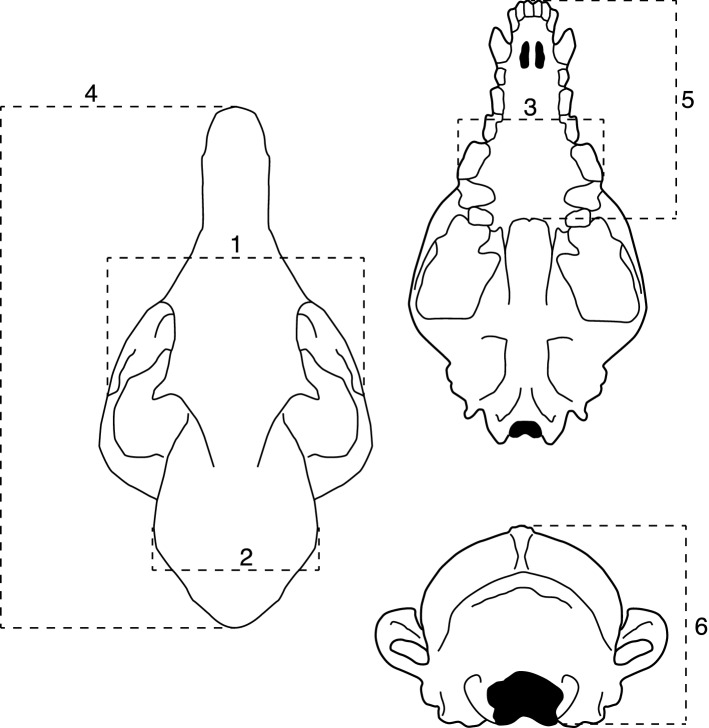
Schematic diagram of the six linear measurements taken on the fox skulls. **1**: Bi-zygomatic width. **2**: Cranial vault width. **3**: Upper jaw width. **4**: Total skull length. **5**: Snout length. **6**: Cranial vault height.

Six linear measurements were taken on each skull using digital calipers (Fowler High Precision, Positional Accuracy ± 0.03 mm): total skull length, snout length, cranial vault height, cranial vault width, bi-zygomatic width and upper jaw width (Fig. 1). Endocranial volume was measured using plastic beads. Each cranium was filled up to the level of the foramen magnum and repeatedly shaken and tamped down until no more beads could be added. The beads were then funneled into a graduated cylinder to obtain a volumetric measurement.

### Geometric morphometrics

Generalized Procrustes analysis was conducted in R v. 4.0.2^18^ using the geomorph v. 3.3.1 package^19^. Landmark configurations from each specimen were translated to the origin, rescaled to centroid size, and optimally rotated (using a least-squares criterion) until the coordinates of homologous landmarks aligned as closely as possible. These steps place all specimens in the same shape space, centered on the mean shape. An orthogonal projection into a linear tangent space was applied so statistical analyses could be performed on the resulting tangent space coordinates. For Procrustes superimposition, we used the default parameters of the gpagen function in geomorph.

### Repeatability analyses

Landmark measurement repeatability was evaluated through repeated measurements of three fox skulls (domesticated male ID# TM23, domesticated female ID# TF476, unselected female ID# UF1058) on ten separate occasions. In this case, repeatability encompasses both Microscribe and operator error. Generalized Procrustes Analysis (GPA) was used on these landmark coordinates to ensure that they were in the same 3D location relative to one another. The average Procrustes distance (PD) between all ten iterations of the same specimen was then compared to the average Procrustes distance within the population-sex grouping to which the skull belonged. To do this, we calculated a sensitivity ratio based on the formula: (Mean Inter-specimen PD – Mean Intra-specimen PD)/Mean Intra-specimen PD. This created a sensitivity ratio that reflects how sensitive the Microscribe measurements are with respect to the average difference among foxes of the same population-sex category. Averaging the sensitivity ratios for our three skulls, we find that the difference between replicates is roughly 3.7 times smaller than the differences within population-sex groupings. This indicates that the Microscribe G2 is robust enough to detect subtle individual differences in measured landmark coordinates. Linear and endocranial measurement repeatability was quantified through a similar method where repeat measurements were taken on 3 domesticated female fox skulls on 15 separate occasions. Sensitivity ratios were deteremined for each measurement (i.e. total skull length, snout length etc.) by calculating the standard deviation of each repeated measurement on a single specimen, averaging the three specimens’ standard deviations for that measurement, and then comparing that value to the population (domesticated female) standard deviation for that measurement. With the exception of cranial vault height (see limitations), the replicate standard deviations of each measurement were roughly a third (or less) of the population standard deviations (Table S3).

### Statistical analyses

All statistical analyses were performed in R^18^. For all parametric inferences, we report point and interval (95% confidence) estimates of effect sizes, while for permutation-based inferences we report point estimates and p-values. All p-values involving multiple comparisons were adjusted for family-wise error using the sequential Bonferroni method.

#### 3D shape comparisons

To test hypotheses about shape differences among the three populations of foxes, we used a permutation-based Procrustes MANOVA to regress tangent space coordinates on population identity and sex in the geomorph v. 3.3.1 package. Because we are unable to detect significant differences in allometry among populations with a permutation-based Procrustes MANOVA of tangent space coordinates on the interaction term of population identity and centroid size, the tangent space coordinates were not corrected for any scaling effects (see Supplemental information and Fig S2). Given this result, we control only for isometric size in geometric morphometric analyses (i.e. no correction for scaling in tangent space coordinates) as well as in our linear measurements. To determine how skull shape differed between fox populations, we performed pairwise comparisons of shape using Procrustes distances. We additionally performed pairwise comparisons between groups of the shape variance within a group (as assessed by the dispersion of residuals around the mean shape for a given population)^20^. All pairwise comparisons were made using the RRPP v. 0.6.1 package^21,22^. Permutation-based p-values for the pairwise comparisons were corrected for family-wise error using the sequential Bonferroni method. To visualize changes in skull shape between populations, a principal components analysis (PCA) was performed on the tangent space coordinates. Skull warp changes along the first principal component were graphed to visualize shape changes along this axis. Size differences between populations were assessed via a linear model using a weighted least squares (WLS) estimator, where centroid size was regressed on population identity and sex. The WLS estimator allowed for separate residual variances for each combination of population and sex, so that heteroskedasticity across these groups could be accounted for in the model. Variance in centroid size was assessed with a Levene's test based on absolute deviations from the median and was performed using the car package v. 3.0-10^23^.

#### Linear and endocranial volume comparisons

Prior to modeling linear and volumetric data, we created size-adjusted versions of our variables to account for a difference in isometric size between wild and RFF populations. Normalizing to size allows us to parse out the effects of size selection from those of selection for docility as they likely have overlapping effects on craniofacial shape. We adjusted for size by normalizing each linear measurement and the cube root of endocranial volume by centroid size. We used centroid size rather than the geometric mean of the six linear measurements because centroid size was calculated using a larger sample of craniometric landmarks and is therefore the better proxy of overall cranial size. We performed size corrections on the raw measurements instead of including a size variable in the models because it allows the size-correction to be intrinsic to each fox rather than depending on the size of every fox in the model.

To determine if there were population-level differences in size-corrected linear and volumetric variables, we used a linear model with a generalized least squares (GLS) estimator from the nlme v. 3.1-150 package^24^ to regress all 7 skull variables simultaneously as correlated responses on population identity and sex (see Supplementary Methods for details of estimation strategy and model specification and Figs. S3, S4). We report estimates of pairwise percent differences between population means for each skull variable. We use this method because the 7 linear and volumetric skull variables were correlated in two ways (see Fig. S3). First, they were measured on the same specimens, and second, they represent non-independent aspects of shape variation. Modeling these response variables in 7 separate general linear models (e.g., ANOVA) would result in biologically unrealistic inferences because these correlations would be artificially fixed at zero. In addition, since skull variables exhibited varying amounts of dispersion, the GLS model allowed for different residual variances for each response variable.

#### Sexual dimorphism comparisons

Sexual dimorphism within a species is often represented as dimorphism in size as well as shape^25^. Therefore, in contrast to the previous analyses, we assess the degree of sexual dimorphism in both size and shape. We used a similar GLS model to determine the degree of sexual dimorphism of the raw (non-size corrected) variables within each population. To estimate sex-specific effects, we added an additional interaction term between sex and population identity in this model. We report the degree of dimorphism using estimates of mean differences between males and females for a given skull variable, within a population. For both models using linear and volumetric data, we performed model selection for variance components and correlation structures using the Bayesian information criterion, since this has been shown to provide a good balance between parsimony and over-fitting for explanatory models^26^. Linear model (GLS) assumptions were checked using diagnostic plots of standardized residuals and fitted values (see Fig. S5).

## Results

### Procrustes analysis and principal components analysis of shape variation

**Figure 2:**
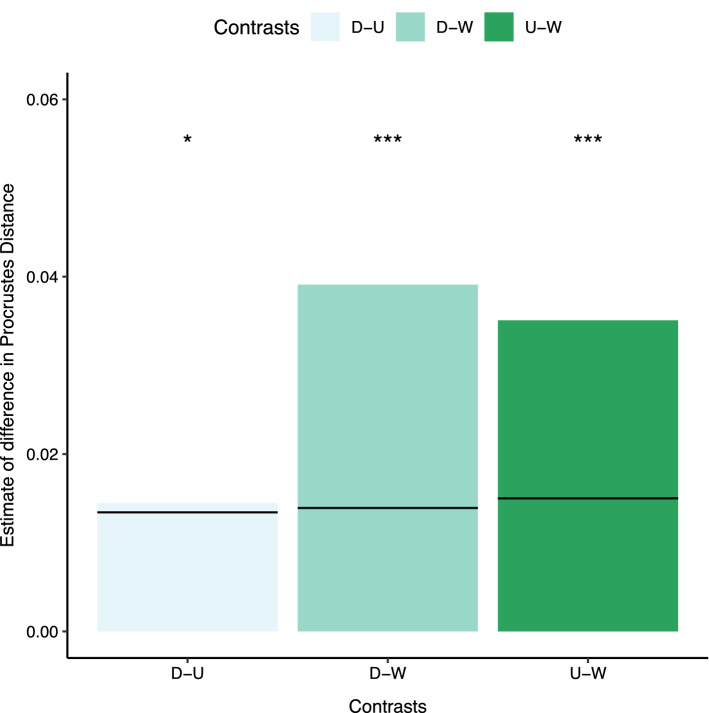
Pairwise comparison of Procrustes distances between populations after a Procrustes MANOVA. D-U: difference between domesticated and unselected populations. D-W: difference between domesticated and wild populations. U-W: difference between unselected and wild populations Significance codes: *p < 0.05, **p < 0.01, ***p < 0.001. Horizontal lines on the graph represent the upper 95% confidence bound of the null hypothesis that the average Procrustes distances between two different populations is 0. Values that lie above these lines indicate a significant difference between the populations indicated in the contrast.

**Figure 3:**
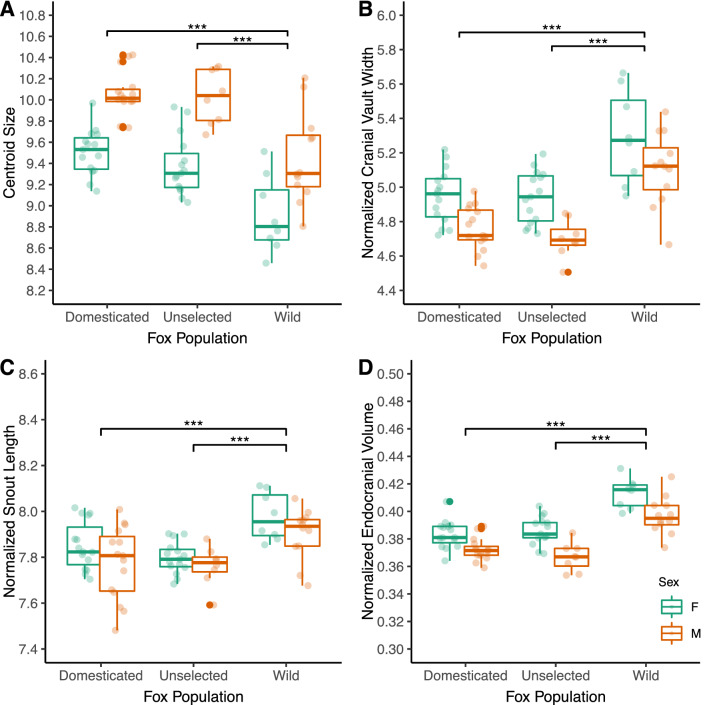
Paired Box and Jitter Plot of selected measurements that differ between the three fox populations. (**A**) Centroid size; (**B**) Normalized cranial vault width; (**C**) Normalized snout length; (**D**) Normalized endocranial volume. ***Represent statistically significant differences between populations as assessed through a linear model with a weighted least squares estimator to account for differences in variances.

Pairwise comparisons, based on a Procrustes MANOVA controlling for sex (Table S4), revealed differences in Procrustes distances between all populations although the difference between wild and either of the RFF populations was approximately three times larger than the difference between RFF populations (D = domesticated, U = unselected, W = wild; estimated mean Procrustes distance difference with the upper 95% confidence bound for the null hypothesis of no difference in Procrustes distance between comparisons and p-values; **D-U**: 0.0145 (0.013), p = 0.028. **D-W**: 0.0391 (0.014), p < 0.001. **U-W**: 0.0351 (0.015), p < 0.001 (Fig. 2). Pairwise comparisons between populations of the magnitude of shape variance within a population found a slight significant difference in the shape variance of the wild and unselected foxes. (Estimated mean shape variance difference with the upper 95% confidence bound for the null hypothesis of no difference in shape variance between comparisons with p-values; **D-U**: 5.5E−5 (1.8E−4), p = 0.58. **D-W**: 1.9E−4 (1.9E−4), p = 0.09. **U-W**: 2.5E−4 (2.0E−4), p = 0.04.) (Table S5). A generalized least squares regression on centroid size controlling for sex found that wild foxes are approximately 5% smaller on the basis of centroid size than both unselected and domesticated populations, which did not differ from one another (Estimated mean difference between centroid sizes with 95% confidence intervals and p-values: **D vs U**: 0.086 (− 0.088, 0.26), p = 0.68; **D vs W**: 0.60 (0.35, 0.85), p < 0.0001; **U vs W**: 0.51 (0.25, 0.78), p = 0.0002) (Fig. 3). Using Levene’s Tests, we were unable to detect any difference in the centroid size variance between populations.

**Figure 4:**
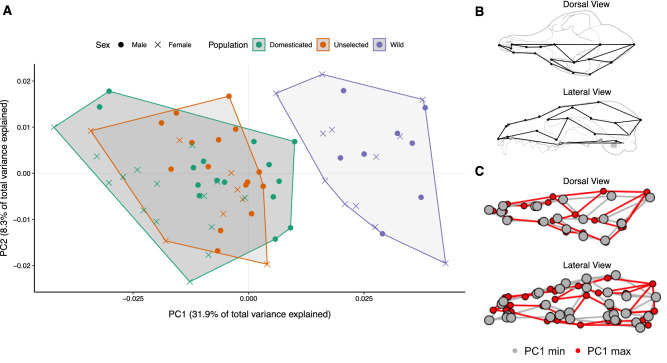
(**A**) Principal Components Analysis of the three fox populations. Scatterplot of the first two principal components of tangent space coordinates from crania of three distinct fox populations: domesticated, unselected, and wild. Note that the PCA plot does not represent overall group differences but is included to show shape variation in PC1 depicted in the wireframes. (**B**) Wireframe overlaid on a schematic fox skull (top: dorsal view; bottom: lateral view). Dots shown in gray correspond to landmarks on the ventral side of the skull. (**C**) Superimposition of the min and max shapes on principal component 1 at two times magnification (top: dorsal view; bottom: lateral view). Note that min and max skull warps do not represent the mean shape of different populations and describe only the variation along PC1.

In the principal components analysis, the first principal component (PC1) accounted for 31.9% of the total variation in skull shape while the second principal component (PC2) only captured about 8.3% of the total variation. Graphing the shape change along PC1 shows a cline of shape change that appears to roughly match the direction of variation from RFF morphs to wild population morphs (Fig. 4). In this cline, those that fall towards the wild end of the spectrum (max values of PC1) possessed skulls with relatively less flexed cranial bases and mediolaterally wider cranial vaults.

### Linear and endocranial volume measurements

We regressed size-normalized linear and volumetric variables on population identity and sex in a linear model using a GLS estimator. Wild foxes differed from both RFF populations in exhibiting relatively wider cranial vaults (~ 7% increase) and marginally longer snout lengths (~ 2% increase). Wild foxes also possessed a larger relative endocranial volume (~ 7% increase or about 3.5 cc) when compared to both unselected and domesticated RFFs (Table 1) (Fig. 3).

**Table 1:** Post-estimation pairwise comparisons of several linear and volumetric measurements among populations after controlling for sex and size in a generalized least squares model.

Trait	Sex-corrected size-adjusted least squares means			Percent differences		
	Domesticated	Unselected	Wild	D-U	D-W	U-W
Zygomatic width	7.34 (7.26, 7.42)	7.41 (7.32, 7.51)	7.31 (7.21, 7.40)	− 1.05% (− 3.7, 1.5)	0.40% (− 2.3, 3.1)	1.45% (− 1.4, 4.3)
Cranial vault width	4.86 (4.79, 4.92)	4.83 (4.76, 4.90)	5.20 (5.13, 5.28)	0.56% (− 2.5, 3.5)	− **6.87% (**− **10.0, **− **3.8)**	− **7.70% (**− **11.2, **− **4.3)**
Upper jaw width	4.44 (4.39, 4.49)	4.48 (4.42, 4.54)	4.46 (4.40, 4.53)	− 0.81% (− 3.6, 1.9)	− 0.51% (− 3.4, 2.3)	0.30% (− 2.8, 3.3)
Total skull length	15.10 (15.04, 15.15)	15.17 (15.10, 15.24)	15.01 (14.94, 15.08)	− 0.49% (− 1.4, 0.4)	0.57% (− 0.4, 1.5)	1.06% (0.0, 2.1)
Snout length	7.81 (7.77, 7.85)	7.78 (7.73, 7.82)	7.94 (7.89, 7.99)	0.47% (− 0.7, 1.7)	− **1.6% (**− **2.9, **− **0.4)**	− **2.09% (**− **3.5, **− **0.7)**
Cranial vault height	4.75 (4.70, 4.80)	4.75 (4.70, 4.81)	4.81 (4.75, 4.86)	− 0.05% (− 2.4, 2.3)	− 1.21% (− 3.7, 1.2)	− 1.16% (− 3.8, 1.4)
Endocranial volume	0.38 (0.37, 0.38)	0.38 (0.37, 0.38)	0.41 (0.40, 0.41)	0.23% (− 2.2, 2.6)	− **6.93% (**− **9.4, **− **4.5)**	− **7.41% (**− **10.2, **− **4.7)**

Least squares means are presented with 95% confidence intervals. Differences between populations are shown as percentage differences with 95% confidence intervals. D-U: difference between domesticated and unselected populations. D-W: difference between domesticated and wild populations. U-W: difference between unselected and wild populations. Differences shown in bold are those that are statistically significant (p < 0.05) after adjustment for family-wise error using the sequential Bonferroni method.

**Figure 5:**
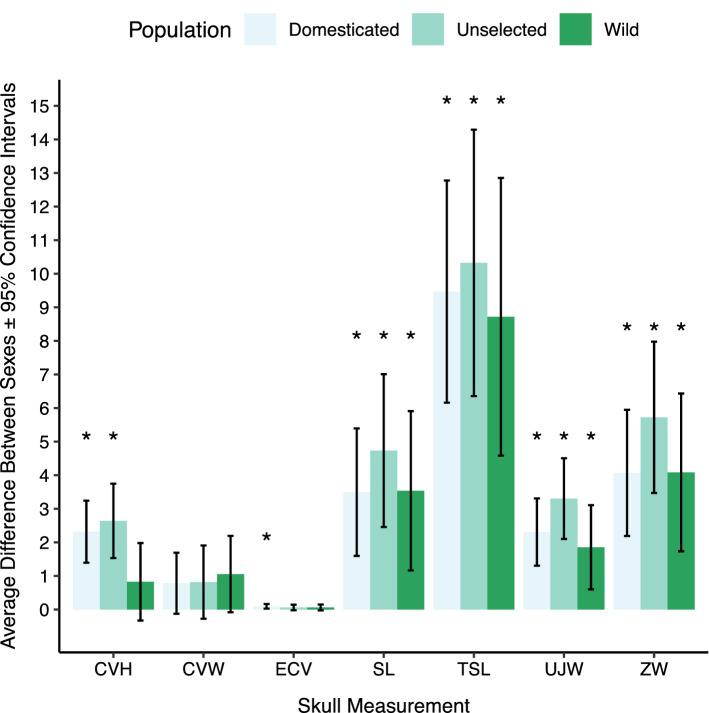
Sexual dimorphism in each raw linear and volumetric measurement as determined by a generalized least squares model. Sexual dimorphism is reported as the average difference between males and females in a population (*CVH* cranial vault height, *CVW* cranial vault width, *ECV* endocranial volume, *SL* snout length, *TSL* total skull length, *UJW* upper jaw width, *ZW* zygomatic width). *Denotes statistically significant difference (P < 0.05) between males and females within a population.

Employing an additional linear GLS model on raw linear and volumetric variables, we found that all populations were sexually dimorphic in most measurements (Fig. 5). Wild foxes exhibited sex-based differences in all measurements except cranial vault width, cranial vault height and endocranial volume, whereas unselected and domesticated RFFs displayed sex differences in every metric except cranial vault width (both) and endocranial volume (only unselected) (Fig. 5).

## Discussion

Although this study must be considered preliminary due to small available sample sizes, our data provide empirical support for the assertion made by Lord and colleagues that substantial changes in the craniofacial morphology of farmed foxes occurred before intense selection for docility was initiated in 1959. Notably, our analyses indicate that the differences between the RFFs and wild populations are greater in magnitude than those between unselected and domesticated RFFs.

When considering the shape data in a Procrustes MANOVA, it is possible to distinguish the three populations based on Procrustes distance. Although all comparisons based on Procrustes distance between the populations are significant, the difference between the wild foxes and each of the farmed populations is greater in magnitude than the difference between the two farmed populations. Since Procrustes distances take all landmarks into account and do not directly address the shape change predictions associated with increased docility, we explored the visualization of shape changes along the first principal component axis, which explains 32% of the shape variation in the study sample. When mapped onto the shape cline represented by PC1, wild skulls tended to exhibit less cranial base flexion and expansion of the cranial vault compared to farmed foxes. Unselected foxes appear to fall between the wild and domesticated individuals in a single direction of shape change. The analysis of linear measurements supports these differences between wild and RFF populations. In contrast, there were no observed differences in linear or volumetric measurements between RFF populations themselves. These results largely agree with the PCA performed in the original morphological study by Trut and colleagues, which shows that when all linear measurements of cranial size were taken into account, RFF populations cannot be distinguished from one another^9^.

The linear and volumetric differences between wild and RFF populations appear to support the predictions of domestication syndrome that have been observed in other domestic animals. The decreases in cranial vault size, snout length and endocranial volume in the RFFs align closely with alterations observed in other domesticates such as dogs and pigs^3,27,28^. While the more flexed cranial bases of the RFF skulls are not normally considered a common marker for domestication syndrome, increased cranial base flexion is a common feature of domestic dogs that is absent in wolves^27^. Despite this, the absence of additional craniofacial change between the RFF populations calls into question the hypothesis that selection for docility is responsible for the craniofacial characteristics ascribed to domestication syndrome.

Our finding that substantial morphological changes occurred before targeted selection for docility suggests that intense selection for docility does not necessarily lead to the craniofacial traits often included in domestication syndrome even if it increases the frequency of rare affiliative behaviors. Instead, some other undocumented evolutionary force associated with captivity that has a greater effect on craniofacial morphology caused the shift in both RFF populations (unselected and domesticated). Many evolutionary pressures could potentially contribute to the craniofacial changes we find between the wild and farmed foxes. Inbreeding and founder effect may be especially important given that the Canadian fur farms started from only a handful of breeding pairs^7^.

Unfortunately, we cannot completely rule out the effects of inadvertent selection for docility on fur farms, which may have partially domesticated the foxes before the RFF experiment began. Unlike their wild counterparts, populations of foxes on fur farms readily breed in captivity and were likely unintentionally selected upon to produce more human affiliative behaviors, such as reduced fear and aggression towards human caretakers^8^. It has recently been shown that foxes living in closer contact with humans (urban environments) possess skulls with relatively wider and shorter snouts than rural-living foxes^29^. These differences in urban fox skulls imply that simply living in closer proximity to humans can produce skull morphologies that are often ascribed to domestication-related changes. That said, it remains unknown if the skull changes are related to dietary differences (different biomechanical challenges or nutrition) or more human-tolerant behaviors in the urban fox populations. Overall, these data in conjunction with the results presented here highlight the benefits of using wild foxes as a comparator group when drawing conclusions from the RFF experiment.

It is notable that the farmed foxes exhibit similar degrees of sexual dimorphism in a number of cranial characteristics when compared to the wild foxes. Although a direct comparison of the degree of sexual dimorphism between populations is not possible with the limited sample size available to us, if there was craniofacial feminization during the process of domestication it was apparently subtle. This fact accords with the lack of craniofacial feminization documented in Belyaev’s selection experiments on rats^30^. However, this is surprising because craniofacial feminization in ‘self-domesticated’ species like bonobos and modern humans^15^ has been used to infer lowered reactive aggression under the assumption that domestication and ‘self-domestication’ lead to similar craniofacial and behavioral outcomes. This assumption therefore needs to be tested further because self-domestication likely involves a more complex set of selection pressures than domestication. Some social selective pressures, like female mate choice and male-male competition, are largely absent during domestication and may lead to different craniofacial phenotypes.

This study has several limitations. The most important is the small sizes available for within species comparisons. Although larger sample sizes would be helpful, our sample size is limited because we want to reconstruct the variation of the progenitor population from which foxes were captured and then kept on Canadian fur farms. Given evidence for regional size and craniofacial shape variation between North American fox subspecies^31,32^, we thus restricted our wild sample to foxes that we knew were trapped in eastern Canada and northern Maine. We also tried to control for temporal variation by looking only at foxes that had been trapped around the beginning of the fur farming industry in Canada. Another limitation on sample size was that many of the foxes from this population in museum collections had sustained trauma to the skull (bullet wounds) from when the animal was dispatched. While our wild sample may not encompass all ancestral variation and future studies may be able to find additional foxes from this population, we believe it is necessary to restrict our wild specimens to ensure that the Fox Farm populations are compared to the best approximation of their progenitor population. We acknowledge that small sample sizes can be problematic in GM analyses if the number of variables exceeds the number of observations, causing spurious separation between groups on discriminatory plots such as bgPCAs and CVAs. However, in our study, this problem is mitigated by correlations between the variables that we measured. In addition, we restrict our metrics of separation to a Procrustes ANOVA which is more tolerant of small sample sizes. The other problem with small sample sizes is that they cannot detect differences below a certain magnitude. Although larger samples are needed to confirm our results, the magnitude of difference between unselected and domesticated foxes, if present, is likely less than the difference observed between wild foxes and the RFFs.

Another limitation in this study is that we were unable to measure dental morphology, a recurring feature of domestication syndrome, due to a large degree of tooth breakage and wear in the wild and unselected populations. In addition, the sensitivity ratio for our measurement of cranial vault height was very low, indicating that this metric may not be reliable. That fact, in addition to our small sample size, means that we are unable to detect any slight variations in skull height between populations from our linear measurements. However, we fail to see these shape changes in the geometric morphometric analysis which indicates that any differences, if present, are subtle.

In sum, we conclude that intense selection for docility in the RFF experiment produced minimal craniofacial change among farmed foxes relative to the craniofacial differences between wild and farmed foxes. Instead, it is reasonable to hypothesize that many craniofacial features ascribed to domestication syndrome arose on Canadian and Russian fur farms. Because numerous selection pressures were acting on those animals, we cannot conclusively prove what caused these changes. More broadly, these results suggest that wild populations should be included as a control group for future studies on genetic and phenotypic alterations associated with domestication, whether in foxes or other species. Additionally, our data emphasize the point that large behavioral shifts, such as greatly increased docility, can have a limited impact on craniofacial morphology. These results suggest that craniofacial skeletal change may be an unreliable marker of the magnitude of behavioral change, and caution against using the skull as a gauge for the amount of behavioral change that occurred within a species, as is commonly done in the hominin fossil record^13,33^.

## Supplementary Information

Supplementary Information

## Data Availability

The data generated by this study are openly accessible in a Zenodo repository (10.5281/zenodo.4265223).
