# Supplementary material for: Geometric morphometric investigation of craniofacial morphological change in domesticated silver foxes
Source: Sci Rep. 2021 Jan 28;11:2582. doi: 10.1038/s41598-021-82111-9 (PMC7843644; doi:10.1038/s41598-021-82111-9)
Supplement: Supplementary file 1 — Supplementary Information [file 41598_2021_82111_MOESM1_ESM.pdf]

# Supplementary Information

## Geometric morphometric investigation of craniofacial morphological change in domesticated silver foxes.

Timothy M. Kistner[1], Katherine D. Zink[1], Steven Worthington[2] and Daniel E. Lieberman[1]*

[1]Department of Human Evolutionary Biology, Harvard University, Cambridge, MA 02138
[2]Institute for Quantitative Social Science, Harvard University, Cambridge, MA 02138

Contact information:

Timothy Kistner — tkistner@g.harvard.edu
Katherine Zink — kzink@oeb.harvard.edu
Steven Worthington — sworthington@iq.harvard.edu
Daniel Lieberman — danlieb@fas.harvard.edu

# Table of Contents

**Supplementary Methods** . . . . . . . . 2
Estimation strategy . . . . . . . . 2
Model specification . . . . . . . . 3
Quantities of interest . . . . . . . . 3
**Supplementary Figures** . . . . . . . . 4
**Supplementary Tables** . . . . . . . . 6
**Supplementary References** . . . . . . . . 8

# Supplementary Methods

## Estimation strategy

We analyzed linear and volumetric measurement data using linear regression with a generalized least squares (GLS) estimator. The GLS estimator of the coefficients of a linear regression is a generalization of the ordinary least squares (OLS) estimator used in the general linear model (GLM). It is used to deal with situations in which the OLS estimator is not the best linear unbiased estimator (BLUE) because one of the main assumptions of the Gauss-Markov theorem, namely that of homoskedasticity and absence of serial correlation, is violated. In such situations, provided that the other assumptions of the Gauss-Markov theorem are satisfied, the GLS estimator is the BLUE. The general linear model is:

$$\vec{y} = \mathbf{X}\vec{\beta} + \vec{\epsilon}$$

where $\vec{y}$ is an $n \times 1$ vector of responses ($n$ is the sample size), $\mathbf{X}$ is a $n \times k$ design matrix of regressors ($k$ is the number of regressors), $\vec{\beta}$ is a $k \times 1$ vector of weights (regression coefficients) to be estimated, and $\vec{\epsilon}$ is an $n \times 1$ vector of errors. We assume that:

1. $\mathbf{X}$ has full rank,
2. $\mathbb{E}[\vec{y} \mid \mathbf{X}] = \mathbf{X}\vec{\beta}$,
3. $\mathbb{E}[\vec{\epsilon} \mid \mathbf{X}] = 0$,
4. $\mathbb{V}\text{ar}[\vec{\epsilon} \mid \mathbf{X}] = \sigma^2\mathbf{I}$, where $\sigma$ is a constant and $\mathbf{I}$ is the $n \times n$ identity matrix.

These are the assumptions made in the Gauss-Markov theorem in order to prove that OLS is the BLUE.[1] Assumption 4 means that the errors of the regression are homoskedastic (they all have the same variance) and uncorrelated (their covariances are all equal to zero). When we use the GLS estimator, we generalize assumption 4 so that:

$$\mathbb{V}\text{ar}[\vec{\epsilon} \mid \mathbf{X}] = \mathbf{V},$$

where $\mathbf{V}$ is an $n \times n$ symmetric positive definite matrix.[1] This allows for heteroskedasticity (the errors can have different variances) and correlation (the covariances between errors can be different from zero):

$$\mathbf{V} = \mathbb{C}\text{ov}\left(\vec{\epsilon_i}, \vec{\epsilon_j}\right) = \mathbb{C}\text{ov}\left(\vec{y_i}, \vec{y_j}\right) = \begin{bmatrix} \sigma^2_{1,1} & \rho_{1,2} & \cdots & \rho_{1,n} \\ \rho_{2,1} & \sigma^2_{2,2} & \cdots & \rho_{2,n} \\ \vdots & \vdots & \ddots & \vdots \\ \rho_{n,1} & \rho_{n,2} & \cdots & \sigma^2_{n,n} \end{bmatrix} .$$

So, the GLS estimator makes *fewer* assumptions than the OLS estimator. If the off-diagonal elements of $\mathbf{V}$ are restricted to be zero (i.e., covariances between errors are not allowed), then we have the weighted least squares estimator (WLS).

In this study, for each specimen, 6 different variables were recorded using linear measurements and 1 variable using volumetric measurement. Our primary interest was to regress each of these variables on population identity and sex. However, analyzing these data by estimating a series of separate GLMs (e.g., ANOVA, ANCOVA, linear regression etc.) would result in biologically unrealistic inferences. This is because the 7 variables are correlated (see Fig. S3) for two related reasons: 1) because they are measured on the same specimens, and 2) because they represent non-independent aspects of shape variation. Since a series of 7 GLMs would involve fitting 7 separate, independent equations, there would be no mechanism to account for the correlations among the responses. Using the GLM approach, the response correlations would be fixed at zero, rather than estimated within the model. The GLS estimator, on the other hand, extends the functionality of the GLM so that the correlations between responses can be estimated and accounted for within a single model. Failing to account for these correlations between responses would result in inferences that are overly optimistic (i.e., interval estimates that are too narrow) and therefore biologically unrealistic.

## Model specification

To include multiple responses in a GLS model framework, the values for each response variable must be 'stacked' on top of one another to form a single response vector $\vec{y}$. The basic explanatory variables are a set of dummy variables (i.e., with values of 0 or 1) that indicate which response variable is present. Further explanatory variables can be included by multiplying these dummy variables (i.e., creating interactions), so that separate effects can be estimated for each response.

In the design matrix $\mathbf{X}$, categorical variables with $g$ levels were converted into $g-1$ dummy variables. The reference level for the $j = 7-1$ 'response' dummy variables (`cranial vault width`, `upper jaw width`, `total skull length`, `snout length`, `cranial vault height`, `endocranial volume`) was set to 'zygomatic width'. The reference level for the $k = 3-1$ 'population' dummy variables (`wild`, `unselected`) was set to 'selected', while for the single 'sex' dummy variable (`male`) it was set to 'female'. The choice of the reference level for all dummy variables is arbitrary and does not change the fitted model, only its parameterization.

Our GLS model specification therefore included 73 specimens and 7 response variables for a total of $n = 73 \times 7 = 511$ observations, with 28 fixed effects parameters, 21 covariance parameters, and 7 variance (error) parameters (the variance-covariance matrix $\mathbf{V}$ was stratified by specimen, so that only observations for a given specimen could covary). For economy of notation, the $j = 7$ individual response variables are referred to here as `response` with a $j$ subscript.

$$
\begin{aligned}
\vec{y}_{ij} = \beta_0 &+ \beta_1 \text{wild}_{ij} \\
&+ \beta_2 \text{unselected}_{ij} \\
&+ \beta_3 \text{male}_{ij} \\
&+ \vec{\beta}_{4-9} \text{response}_j \\
&+ \vec{\beta}_{10-15} \text{response}_j \times \text{wild}_{ij} \\
&+ \vec{\beta}_{16-21} \text{response}_j \times \text{unselected}_{ij} \\
&+ \vec{\beta}_{22-27} \text{response}_j \times \text{male}_{ij} \\
&+ \vec{\epsilon}_{ij} \quad ,
\end{aligned}
$$

where $\vec{y}_{ij}$ is a vector of $i = 73$ specimens for each of $j = 7$ responses, $\beta_1$ is the expected difference between wild and selected foxes for response $j = 1$ (`zygomatic width`), $\beta_2$ is the expected difference between unselected and selected foxes for response $j = 1$, $\beta_3$ is the expected difference between male and female foxes for response $j = 1$, $\vec{\beta}_{4-9}$ is a vector of effects for selected foxes, $\vec{\beta}_{10-15}$ is a vector of effects for selected versus wild foxes, $\vec{\beta}_{16-21}$ is a vector of effects for selected versus unselected foxes, $\vec{\beta}_{22-27}$ is a vector of effects for female versus male foxes, and $\vec{\epsilon}$ is a vector of errors for each combination of specimen and response. We can visualize this model specification using a path diagram (see Fig. S4). In addition, when testing hypotheses related to sexual dimorphism we extended the above specification to include the 3-way interaction (and all subordinate terms) between `response`, `male`, `wild`, and `unselected`.

For models using a GLS or WLS estimator, we used the Bayesian information criteron (BIC) to select models with an appropriate variance-covariance structure for $\mathbf{V}$. The BIC is a measure of goodness-of-fit that penalizes model complexity and is typically used for explanatory models to provide a balance between parsimony and over-fitting.[2] Model assumptions were checked using diagnostic plots of standardized residuals versus fitted values and sample quantiles versus Gaussian quantiles (see Fig. S5).

## Quantities of interest

We extracted quantities of interest, such as predicted marginal means and marginal effects, from the fitted model using linear combinations of coefficients in the `R` package `emmeans` v. 1.5.1.[3] The estimands of particular interest to us were the predicted marginal means of population identity and sex for each response variable and the pairwise marginal effects between these means. Point and interval (95% confidence) estimates of each size normalized effect size are reported.

## Supplementary Figures

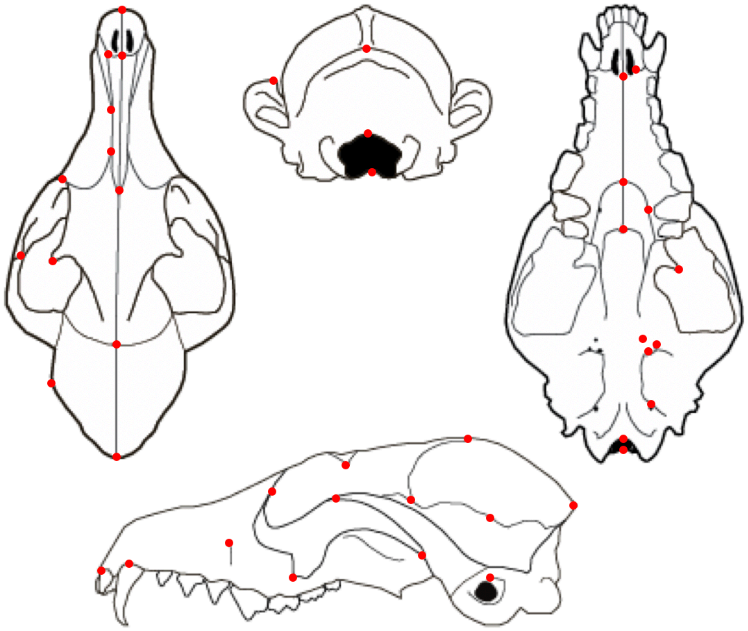

**Figure S1:** Locations of landmarks used in this study. Left: dorsal view, Top: posterior view, Right: ventral view, Bottom: lateral view (not all landmarks are shown on each view).

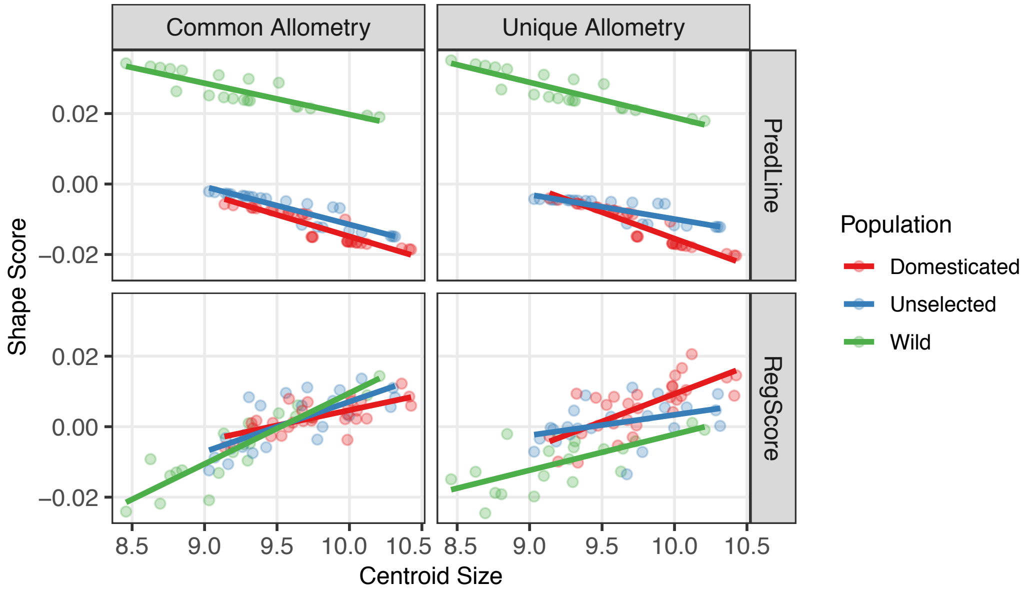


**Figure S2:** Scatterplots of shape scores by centroid size. Left panels are generated from a model of common allometry (i.e., different population means but same slope) while right panels are generated from a model of unique allometry (i.e., different population means and slopes). Upper panels display PredLine shape scores while lower panels display RegScore shape scores.

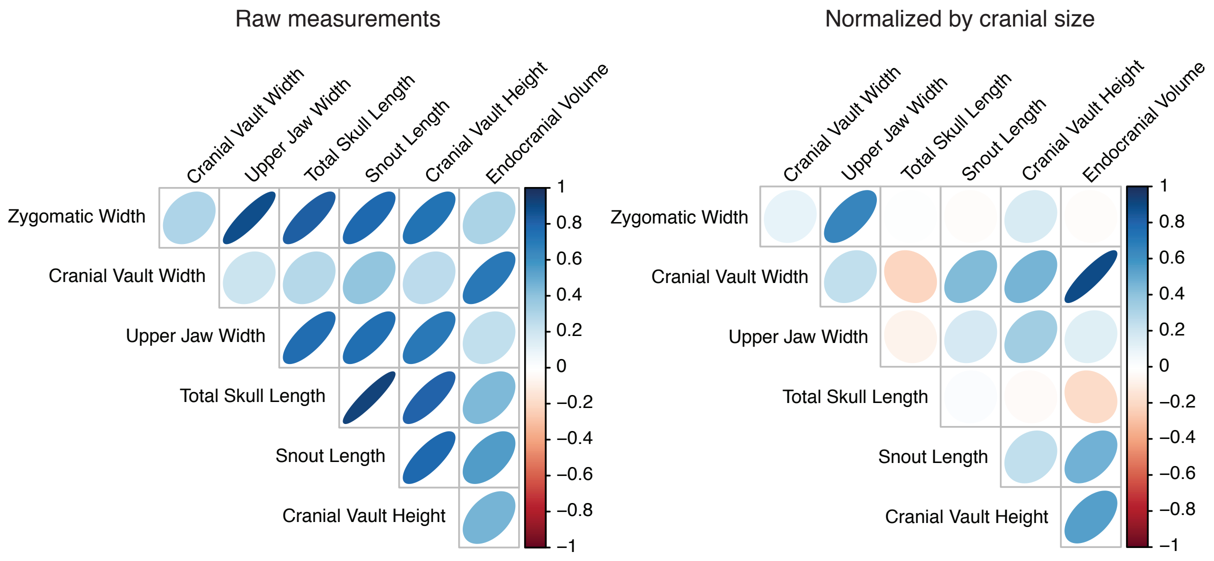


**Figure S3:** Correlation matrices of 7 response variables. Left panel: raw variables. Right panel: size normalized variables (denominator is centroid size).

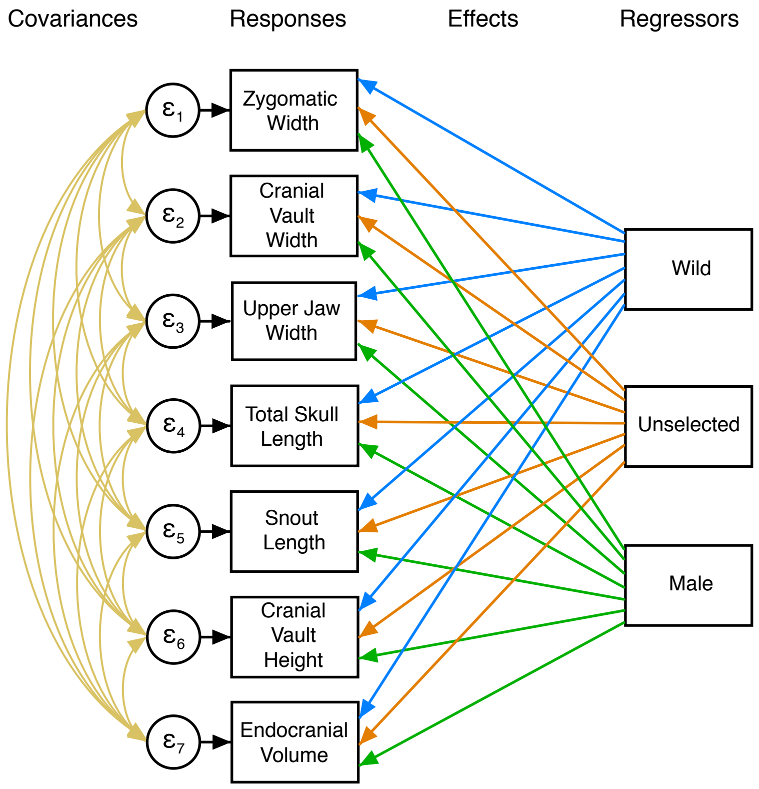


**Figure S4:** Path diagram of GLS model specification.

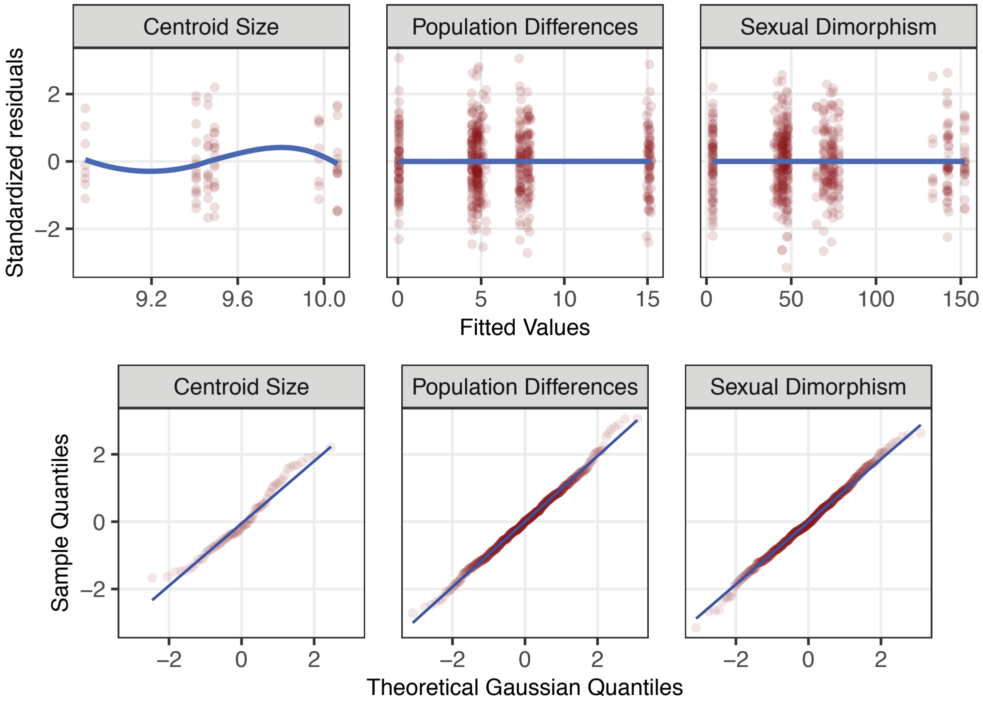


**Figure S5:** Diagnostic plots of model fits for the 3 models using linear and volumetric variables. Top panels: red points are standardized residuals by fitted values, while blue lines are locally weighted (loess) smoothers. Bottom panels: red points are sample quantiles by Gaussian quantiles, while blue lines represent the idealized one-to-one relationship.

# Supplementary Tables

**Table S1:** Trap year and location of wild fox samples. The exact collection year of one specimen `MCZ60057` could not be determined from museum records. In the case of this specimen, 1987 is the latest that this individual could have been trapped and it was likely trapped before then.

| Catalog Number | Location | Trap Year |
|---|---|---|
| *Females* | | |
| `MCZ1178` | Newfoundland | 1894 |
| `MCZ1992` | Nova Scotia | 1894 |
| `MCZ3793` | Newfoundland | 1895 |
| `MCZ3794` | Newfoundland | 1895 |
| `MCZ3795` | Newfoundland | 1895 |
| `MCZ7471` | Labrador | 1900 |
| `MCZ42388` | Quebec | 1945 |
| `MCZ31933` | New Brunswick | 1931 |
| *Males* | | |
| `MCZ1991` | Nova Scotia | 1894 |
| `MCZ2001` | Nova Scotia | 1894 |
| `MCZ3792` | Newfoundland | 1895 |
| `MCZ4299` | New Brunswick | 1896 |
| `MCZ6952` | Ottawa | 1897 |
| `MCZ8965` | Labrador | 1899 |
| `MCZ8963` | Labrador | 1898 |
| `MCZ3791` | Newfoundland | 1895 |

| Catalog Number | Location | Trap Year |
|---|---|---|
| MCZ42508 | Quebec | 1946 |
| MCZ47485 | Quebec | 1952 |
| MCZ60057 | Maine | ? |
| MCZ52832 | Maine | 1948 |

**Table S2:** List of landmark definitions used in 3D geometric morphometric analyses. * denotes that this landmark was measured at the midline of the skull. Type 1 landmarks are supported by histology, while type 2 are supported by geometry. Type 3 landmarks are those with at least one deficient coordinate.

| Name | # | Description | Type |
|---|---|---|---|
| Prosthion | 1 | The most anterior point of the maxillary alveolar process (measured at the first incisor on the left side) | 2 |
| Nasal / Premaxillary | 2 | The most anterior point of the nasal and premaxillary suture | 2 |
| Rhinion | 3 | The most anterior point of the internasal suture* | 2 |
| PMN Suture | 4 | Intersection of premaxilla, maxilla, and nasal sutures | 1 |
| FMN Suture | 5 | Intersection of frontal, maxilla, and nasal sutures | 1 |
| Nasion | 6 | Intersection of the internasal and fronto-nasal sutures* | 1 |
| Bregma | 7 | Intersection of the coronal and sagittal sutures* | 1 |
| Inion | 8 | Intersection of the nuchal and sagittal crests* | 1 |
| Canine Alveolar | 9 | Midpoint of canine width measured at the lateral margin of the alveolar process | 2 |
| Infraorbital foramen | 10 | Most dorsal point on the infraorbital foramen | 2 |
| Orbtial Rim | 11 | Intersection of the frontal and zygomatic bones on the orbital rim | 1 |
| Ant. Jugal | 12 | The most anterior portion of the jugal bone ventral to the notch in the jugal-maxillary suture | 2 |
| Dorsal Jugal | 13 | The most dorsal portion of the jugal bone and the anterior point of the suture between jugal and squamosal | 2 |
| Post Orbital Process | 14 | The lateral most point of the post orbital process | 2 |
| Pterion | 15 | Intersection of parietal, squamosal, and frontal bones | 1 |
| Post. Jugal | 16 | The most posterior point of the jugal bone and the posterior point of the suture between jugal and squamosal | 2 |
| Ext. Auditory Meatus | 17 | The most dorsal point of external auditory meatus | 2 |
| Euryon | 18 | The widest diameter of the cranial breadth measured on the border between parietal and temporal bones | 3 |
| Max / Premax | 19 | The premaxilla-maxilla suture taken along the lateral border of the incisive foramen | 2 |
| Incisive | 20 | Midpoint tangent to the most posterior margin of the incisive foramen* | 2 |
| Palatine | 21 | Intersection between palatomaxillary suture and median palatine suture* | 1 |
| Staphylion | 22 | Midpoint tangent to the palatine notches* | 2 |
| Rotundum | 23 | Most anterior point of foramen rotundum | 2 |
| Ovale | 24 | Most posterior point of foramen ovale | 2 |
| Lacerum | 25 | Most posterior point of foramen lacerum | 2 |
| Hypoglossal | 26 | Most posterior point of the hypoglossal foramen | 2 |
| Basion | 27 | The most anterior point of the foramen magnum* | 2 |
| Opisthion | 28 | The most posterior point of the foramen magnum* | 2 |
| Palatine Foramen | 29 | The most posterior point of the palatine foramen | 2 |

**Table S3:** Measurement repeatability analyses on individual craniofacial variables. Intra-specimen standard deviation (SD) represents the average SD of 15 separate measurements across three different domesticated female fox skulls. Inter-specimen SD represents the SD across the domesticated females in the entire study sample. The sensitivity ratio is calculated as follows: Inter-specimen SD − Intra-specimen SD/Intra-specimen SD. The ratio gives a rough estimate of the magnitude of difference between the intra-specimen SD and inter-specimen SD, with larger numbers indicating a more precise measurement.

| Variable | Intra-specimen SD | Inter-specimen SD | Sensitivity Ratio |
|---|---|---|---|
| Zygomatic Width (mm) | 0.57 | 1.63 | 1.86 |
| Cranial Vault Width (mm) | 0.12 | 1.37 | 10.42 |
| Upper Jaw Width (mm) | 0.13 | 1.00 | 6.69 |
| Total Skull Length (mm) | 0.44 | 3.48 | 6.91 |
| Snout Length (mm) | 0.13 | 1.81 | 12.92 |
| Cranial Vault Height (mm) | 0.59 | 0.94 | 0.59 |
| Endocranial Volume (cc) | 0.73 | 3.56 | 3.88 |

**Table S4:** Procrustes MANOVA table with sex and population as predictors. df: degrees of freedom. SS: sums of squares. MS: mean squares. $R^2$: partial R-squared. F: F-values. Z: standard deviates of sampling distributions of F-values.

| Model term | df | SS | MS | $R^2$ | F | Z | P-value |
|---|---|---|---|---|---|---|---|
| Sex | 1 | 0.003 | 0.003 | 0.03 | 3.3 | 4.0 | <0.0001 |
| Population | 2 | 0.023 | 0.012 | 0.26 | 12.8 | 8.3 | <0.0001 |
| Residual | 69 | 0.063 | 0.001 | 0.71 | | | |
| Total | 72 | 0.088 | | | | | |

**Table S5:** Pairwise comparisons of shape variance (dispersion of residuals) around mean population shape. D: domesticated, U: unselected, W: wild. d: Effect size expressed as differences between population variances (first population minus second population). UCL (95%): 95% upper bound confidence limit for the null hypothesis of no difference in shape variance between populations. Z: Z-score of the variance of the second population with respect to the variance of the first population. P-values have been adjusted for family-wise error using the sequential Bonferroni method.

| Comparison | d | UCL(95%) | Z | P-value |
|---|---|---|---|---|
| D vs U | 0.000055 | 0.00018 | -0.39 | 0.58 |
| D vs W | 0.000195 | 0.00019 | 1.98 | 0.089 |
| U vs W | 0.000250 | 0.00020 | 2.70 | 0.039 |

# Supplementary References


1. Cameron, A. C. & Trivedi, P. K. *Microeconometrics: Methods and Applications.* (Cambridge University Press, 2005).

2. Schwarz, G. Estimating the dimension of a model. *The Annals of Statistics* **6**, 461–464 (1978).

3. Lenth, R. *Emmeans: Estimated marginal means, aka least-squares means. R package version 1.5.1.* (2020).
